# Drug-eluting beads transcatheter arterial chemoembolization combined with systemic therapy versus systemic therapy alone as first-line treatment for unresectable colorectal liver metastases

**DOI:** 10.3389/fonc.2024.1338293

**Published:** 2024-04-24

**Authors:** Fuquan Wang, Lei Chen, Chai Bin, Yanyan Cao, Jihua Wang, Guofeng Zhou, Chuansheng Zheng

**Affiliations:** ^1^ Department of Radiology, Union Hospital, Tongji Medical College, Huazhong University of Science and Technology, Wuhan, China; ^2^ Hubei Province Key Laboratory of Molecular Imaging, Wuhan, Hubei, China

**Keywords:** colorectal liver metastases, drug-eluting beads transarterial chemoembolization, combination therapy, progression-free survival, treatment response

## Abstract

**Purpose:**

The purpose of this retrospective study was to compare the therapeutic efficacy and safety of drug-eluting bead transarterial chemoembolization (DEB-TACE) combined with systemic therapy to systemic therapy alone as first-line treatment for unresectable patients with colorectal liver metastases (CRLM).

**Methods:**

From December 2017 to December 2022, patients with unresectable CRLM who received systemic therapy with or without DEB-TACE as first-line treatment were included in the study. The primary endpoint was progression-free survival (PFS). Secondary endpoints were tumor response, conversion rate and adverse events.

**Results:**

Ninety-eight patients were enrolled in this study, including 46 patients who received systemic therapy combined with DEB-TACE (DEB-TACE group) and 52 patients who received systemic therapy alone (control group). The median PFS was elevated in the DEB-TACE group compared with the control group (12.1 months vs 8.4 months, *p* = 0.008). The disease control rate was increased in the DEB-TACE group compared with the control group (87.0% vs 67.3%, *p* = 0.022). Overall response rates (39.1% vs 25.0%; *p* = 0.133) and conversion rate to liver resection (33.8% vs 25.0%; *p* = 0.290) were no different between the two groups. The multivariate analysis showed that treatment options, size of liver metastasis, number of liver metastasis, synchronous metastases, and extrahepatic metastases were independent prognostic factor of PFS. Further subgroup analyses illustrated that PFS was beneficial with the DEB-TACE group in patients with age ≥ 60, male, left colon, synchronous metastases, bilobar, number of liver metastasis > 5, extrahepatic metastases, non-extrahepatic metastases, CEA level < 5 (ng/ml), and KRAS wild-type. No grade 4 or 5 toxicities related to DEB-TACE procedures were observed.

**Conclusion:**

In patients with unresectable CRLM, systemic chemotherapy with DEB-TACE as first-line treatment may improve progression-free survival and disease control rate outcomes over systemic chemotherapy alone with manageable safety profile.

## Introduction

Liver metastasis is a significant determinant of the prognosis for colorectal cancer (CRC) patients, often resulting in organ failure and a high mortality rate (1, 2). Over 80% of patients diagnosed with colorectal liver metastases (CRLM) are not suitable candidates for surgical removal (3). Typically, patients with unresectable CRLM administered systemic FOLFOX (folinic acid, 5-fluorouracil, oxaliplatin) or FOLFIRI (folinic acid, 5-fluorouracil, and irinotecan) treatment, either with or without bevacizumab or cetuximab as first-line therapy (4, 5). However, the effectiveness of this approach is moderate, with only a 50 percent response rate (6). Hence, the clinical treatment of CRLM focuses on strengthening the therapeutic effect of first-line treatment. Regional therapies for liver metastases, such as transarterial chemoembolization (TACE), hepatic artery infusion chemotherapy (HAIC), and transarterial radioembolization (TARE), are considered alternative optional treatments for patients with unresectable CRLM (7).

TACE is a localized therapy that delivers chemotherapeutic drugs to the tumor area through the hepatic artery, with both cytotoxic effects against metastases and embolization of the feeding arteries of the tumor (8). Drug-eluting beads transcatheter arterial chemoembolization (DEB-TACE), an advancement on conventional TACE, applies microspheres loaded with drugs for permanent embolization and allows for a more consistent and steady release of drugs, in turn decreasing the side effects and improving the therapeutic result (9, 10). Reported preoperative DEB-TACE could improve recurrence-free survival and overall response rates (ORR) with limited adverse events in patients with CRLM undergoing conversional hepatectomy (9). As a palliative treatment, DEB-TACE could also benefit the survival of patients with unresectable CRLM who had failed systemic chemotherapy (11–13). Moreover, previous studies demonstrated that DEB-TACE plus systemic chemotherapy as second-line treatment exhibited encouraging progression-free survival (PFS) outcomes for patients with unresectable CRLM (14, 15). Consequently, we hypothesize that DEB-TACE combined with systematic chemotherapy as first-line treatment could potentially be more efficacious than systemic chemotherapy alone.

Therefore, the purpose of this study was conducted to evaluate the efficacy and safety of DEB-TACE combined with systematic chemotherapy versus chemotherapy alone as first-line treatment in patients with unresectable CRLM.

## Materials and methods

### Patients

From December 2017 to December 2022, unresectable CRLM patients who underwent systemic therapy with or without DEB-TACE treatment from the Wuhan Union Hospital were involved in our study. The inclusion criteria were as follows: (1) patients diagnosed with colorectal cancer liver metastases confirmed histologically; (2) patients treated by systemic therapy with or without DEB-TACE treatment; (3) Eastern Cooperative Oncology Group (ECOG) performance status scores of 0 or 1; (4) aged >18 years. The exclusion criteria were as follows: (1) prior treatment with hepatectomy, radiation, or thermoablation of the liver; (2) liver metastases burden exceeding 70% of liver volume; (3) severe hepatic failure or renal impairment; (4) patients with incomplete medical records.

The study was approved by the institutional review board of our local hospital. The requirement of informed consent was waived due to the retrospective study design.

### Treatment

In the DEB-TACE group (DEB-TACE plus systemic chemotherapy), a treatment course included one DBE-TACE procedure and three standard cycles of chemotherapy. DEB-TACE was performed on day one, followed by chemotherapy on day 14. In the control group (systemic chemotherapy alone), a treatment course involved four cycles of systemic therapy over eight weeks. The treatment course continued until metastases progression or death. The chemotherapy schedule included FOLFOX (oxaliplatin 85 mg/m^2^ and folinic acid 400 mg/m^2^ as a 2-h infusion at day 1, and 5-FU 400 mg/m^2^ intravenous at day 1 and 2400 mg/m^2^ 46-h continuous infusion) or FOLRIRI (irinotecan 180 mg/m^2^ and folinic acid and 5-FU at day 1, administered as for mFOLFOX6). Cetuximab 400 mg/m^2^ and thereafter 250 mg/m^2^ were administered once per or every other week. Bevacizumab 5 mg/kg was administered every other week.

The DEB-TACE operations were performed under the supervision of several experienced interventional radiologists. The Seldinger technique was performed under local anesthetics. The transfemoral arterial access route was constructed through a 5-F 12-cm sheath (Terumo, Tokyo, Japan). After that, a 5-F catheter (COOK, IN, USA) was introduced to the common hepatic artery and superior mesenteric artery to distinguish the tumor-supplying artery. When confirming the artery, a 2.7-F coaxial microcatheter (Progreat, Terumo, Tokyo, Japan) was used to perform super-selective arterial catheterization. Then, the 80 mg irinotecan loaded into one vial (2 mL) beads (CalliSpheres^®^ microspheres, Hengrui Medicine, Inc., Jiangsu, China) mixed with non-ionic contrast agents were delivered via the microcatheter. Until the angiography showed that the staining of the tumor disappeared or almost disappeared, the operation ceased. When further embolization was required, Polyvinyl Alcohol (PVA) was used to perform it until near-stasis.

Patients were evaluated by imaging after each course of treatment, and repeated TACE procedures were determined according to the degree of treatment response and quality of life. Physicians decided to stop TACE procedures when the tumor converted to resectable or the tumor decreased enough to avoid the risk associated with liver disease, or the quality of life might be reduced due to repeated hospitalization. After the termination of TACE treatment, systemic therapy alone continued every two weeks until the tumor progressed. Conversion to resectability required evaluation by a specialized surgeon in accordance with established standards for resectability as reported.

### Follow-up and assessment criteria

All patients were followed up until 31 December 2022. Abdominal contrast-enhanced MRI or CT, chest and pelvis-enhanced CT, and laboratory were carried out before treatment and every treatment course (8 weeks). Progression-free survival (PFS) served as the primary endpoint. Overall response rate (ORR), disease control rate (DCR), conversion rate to liver resection, and adverse events were used as secondary objectives.

Both overall PFS and liver-specific PFS were evaluated. The treatment response including complete response (CR), partial response (PR), stable disease (SD), and progressive disease (PD), was evaluated according to the Response Evaluation Criteria in Solid Tumors 1.1 protocol (RECIST 1.1) (16). ORR was referred to the percentage of patients who attained CR or PR. DCR was defined as the sum of CR, PR, and SD. All adverse events during and post-treatment were noted and graded according to the Common Terminology Criteria for Adverse Events (version 5.0).

### Statistical Analysis

The SPSS software (version 25.0; IBM, San Jose, New York, USA) and R software (version 4.1.1; Vienna, Austria) were applied to fundamental statistical analysis. Data are expressed in the count (%), median, or mean ± standard deviation. Continuous variables were calculated by Student’s t-test, and categorical variables were compared using Fisher’s exact test or the chi-squared test. Kaplan-Meier curves were plotted to assess patients’ PFS, while a log-rank test was employed to compare the two groups. Univariate analysis was applied, while the factors with *p*-value<0.1 were further assessed by multivariate analysis to confirm the potential prognostic factors impacting PFS. *p* value <0.05 (two-tailed) was regarded as statistically significant.

## Results

### Treatment characteristics

**Figure 1:**
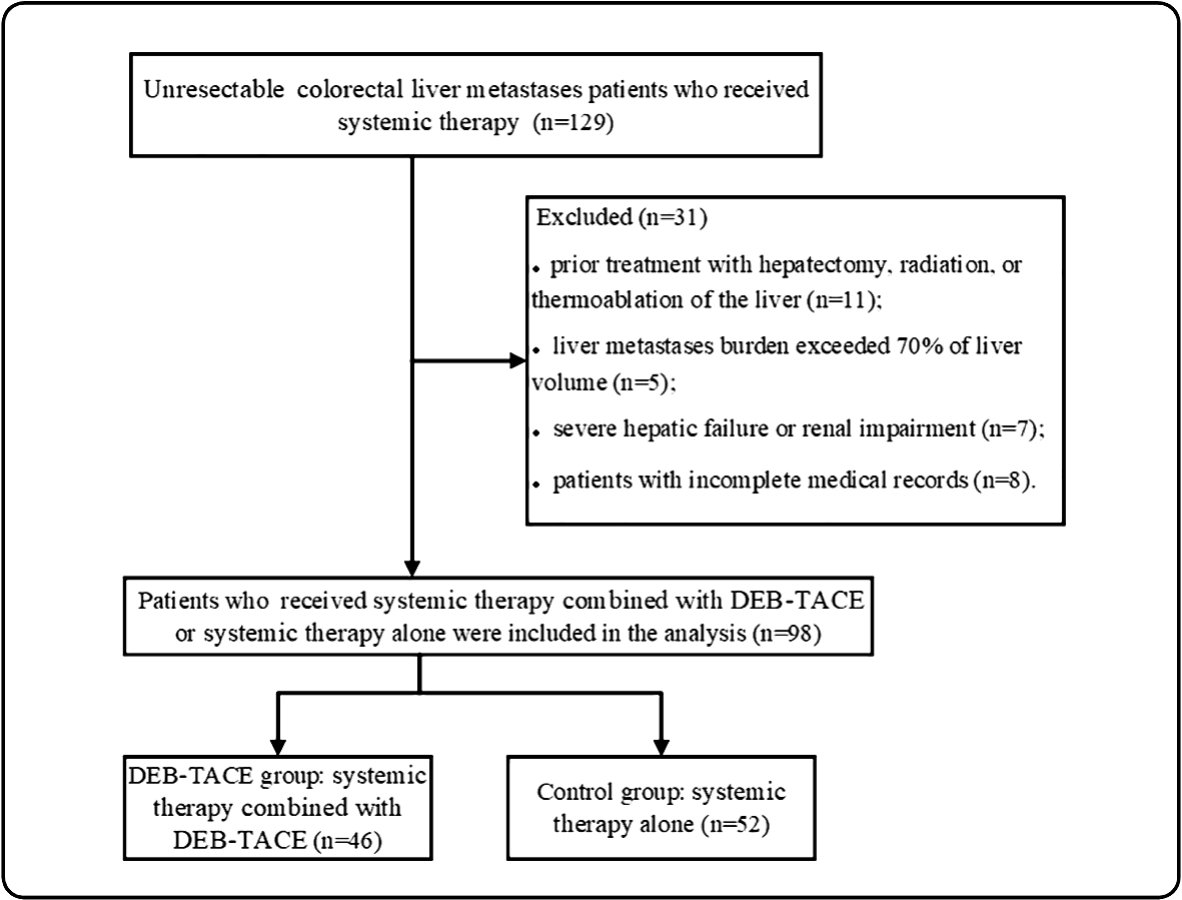
Patients enrolment flow chart. DEB-TACE, drug-eluting bead transarterial arterial chemoembolization.

From December 2017 to December 2022, 129 unresectable CRLM patients were recruited in our study. Ninety-eight patients met the criterion of acceptability in this analysis: 46 patients received DEB-TACE combined with systemic therapy (DEB-TACE group), and 52 patients were treated with systemic therapy alone (control group) (**Figure 1**). The baseline characteristics of the 98 patients with unresectable CRLM are summarized in **Table 1**. The baseline characteristics did not differ between groups. The median follow-up period was 14 months (range, 2–33 months) in the DEB-TACE group and 10 months (range, 2–28 months) in the control group.

**Table 1:** Baseline characteristics of patients.

Characteristic	DEB-TACE (n=46) (No, %; Mean ± SD)	Control (n=52) (No, %; Mean ± SD)	*p* value
**Gender**			0.400
Male	30 (65.2%)	38 (73.1%)	
Female	16 (34.8%)	14 (26.9%)	
**Age (years)**	56.7 ± 10.0	59.5 ± 10.8	0.358
**Primary site**			0.948
Rectum	17(37.0%)	20(38.5%)	
Left colon	18(39.1%)	21(40.4%)	
Right colon	11(23.9%)	11(21.2%)	
**Primary tumor resected**			0.672
Yes	37(80.4%)	40(76.9%)	
No	9(19.6%)	12(23.1%)	
**Synchronous metastases**			0.418
Yes	36 (78.3%)	44 (84.6%)	
No	10 (21.7%)	8 (15.4%)	
**Size of liver metastasis**	2.67 ± 1.51	2.77 ± 1.60	0.746
**Number of liver metastasis**			0.863
≤ 5	14(30.4%)	15(28.8%)	
> 5	32(69.6%)	37(71.2%)	
**Location of liver metastases**			0.337
bilobar	35 (76.1%)	35 (67.3%)	
unilobar	11 (23.9%)	17 (32.7%)	
**Extrahepatic Metastases**			0.814
Yes	8 (17.4%)	10 (19.2%)	
No	38 (82.6%)	42 (80.8%)	
**CEA level (ng/ml**)			0.993
≤ 5	15 (32.6%)	17 (32.7%)	
> 5	31 (67.4%)	35 (67.3%)	
**KRAS type**			0.749
Wild	18 (39.1%)	22 (42.3%)	
Mutated	28 (60.9%)	30 (57.7%)	
**NRAS type**			1.0
Wild	45 (97.8%)	50 (96.2%)	
Mutated	1 (2.2%)	2 (3.8%)	
**BRAF type**			1.0
Wild	45 (97.8%)	51 (98.1%)	
Mutated	1 (2.2%)	1 (1.9%)	
**First-line chemotherapy**			0.969
FOLFOX	4 (8.7%)	6 (11.5%)	
FOLFOX + bevacizumab	12 (26.1%)	15 (28.8%)	
FOLFOX + cetuximab	8 (17.4%)	11 (21.2%)	
FOLFIRI	3 (6.5%)	2 (3.8%)	
FOLFIRI + bevacizumab	5 (10.9%)	4 (7.7%)	
FOLFIRI + cetuximab	4 (8.7%)	3 (5.8%)	
Other	10 (21.7%)	11 (21.2%)	

FOLFOX, folinic acid, 5-fluorouracil (5-FU), oxaliplatin; FOLFIRI, folinic acid, 5-FU, irinotecan.

### Treatment efficacy

The median number of treatment cycles was 5 in the DBE-TACE group and 4 in the control group. Imaging assessment information with RECIST, version 1.1, response was conducted at 6 months plus or minus 2 weeks after treatment. In the DBE-TACE group, 18 out of 46 patients (39.1%) met PR criteria, 22 patients (47.9%) achieved SD, 6 patients (13.0%) were PD, and there were no CR occurred (**Table 2**). In the control group, 13 (25.0%) patients met PR criteria, 22 patients (42.3%) achieved SD, 17 patients (32.7%) were PD, and also no CR occurred. The ORR of tumor response was 39.1% in the DEB-TACE group and 25.0% in the control group, with no significant difference (*p* = 0.133). It is worth mentioning that the DCR was significantly higher with the DEB-TACE group compared to the control group (87.0% vs 67.3%, *p* = 0.022). The new extrahepatic disease was seen in 4 (8.7%) of the DEB-TACE group and 8 (15.4%) of the control group, albeit with no significant difference observed (*p* = 0.313).

**Table 2:** Treatment efficacy.

Parameter	DEB-TACE (n=46)	Control (n=52)	*p* value
**Partial response**	18 (39.1%)	13 (25.0%)	0.133
**Stable disease**	22 (47.9%)	22 (42.3%)	0.584
**Progressive disease**	6 (13.0%)	17 (32.7%)	0.022
**Overall response rate**	18 (39.1%)	13 (25.0%)	0.133
**Disease control rate**	40 (87.0%)	35 (67.3%)	0.022
**Conversion to resection**	16 (33.8%)	13 (25.0%)	0.290
**Extrahepatic progress**	4 (8.7%)	8 (15.4%)	0.313

**Figure 2:**
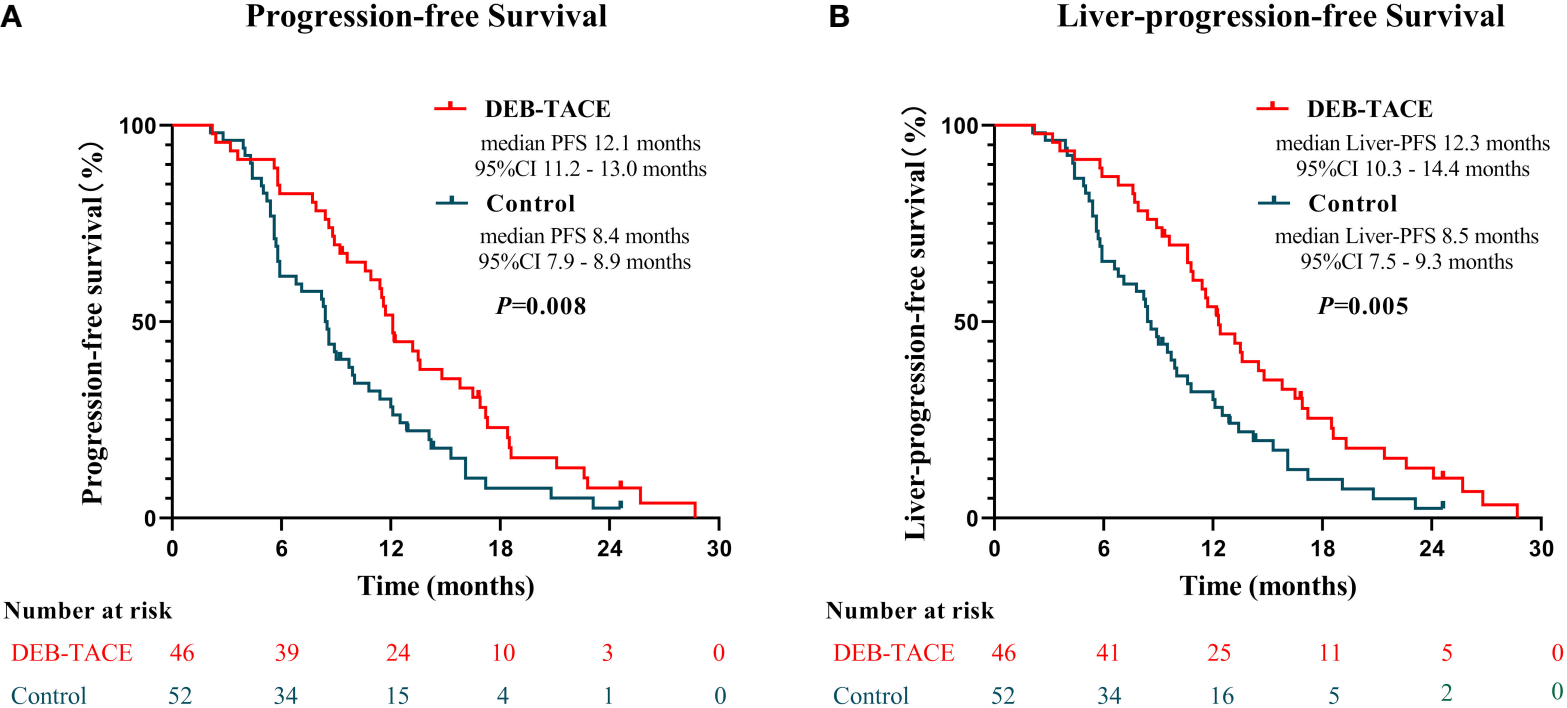
Progression-free survival **(A)** and Liver-progression-free survival **(B)** of patients in the control group and the DEB-TACE group.

The median PFS was elevated in the DEB-TACE group (12.1 months, 95% confidence interval [CI]:11.2, 13.0 months) compared with the control group (8.4 months, 95% CI:7.9, 8.9 months) (*p* = 0.008). Meanwhile, the median liver-PFS was also significantly longer in the DEB-TACE group than in the control group (12.3 months [95% CI, 10.3–14.3 months] vs 8.5 months [95% CI, 7.5–9.3 months], *p* = 0.005) (**Figure 2**). The half-year PFS was 84.8% in the DEB-TACE group, higher than the 65.4% in the control group (*p* = 0.028). Furthermore, the one-year PFS was also significantly higher in the DEB-TACE group than in the control group (52.2% vs 28.8%, *p* = 0.019). The conversion rate to liver resectable was 33.8% in the DEB-TACE group, while the control group had 25.0%, with no statistically significant difference (*p* = 0.290). The median PFS of these patients who were converted to resectability was 13.5 months, which was higher than that of patients who were unresectable in 8.5 months (*p* < 0.001).

### Prognostic factors for PFS

The independent factors predictive of PFS based on univariate and multivariate Cox regression analyses are summarized in **Table 3**. The univariant analysis demonstrated that the therapy method (DEB-TACE plus systemic treatment vs systemic treatment alone) was associated with PFS in CRLM patients (hazard ratio [HR], 0.569; 95%CI: 0.372-0.871; *p* = 0.010). After adjustment by multivariant regression, the treatment method could be an independent factor in predicting PFS in CRLM patients (HR, 0.482; 95%CI: 0.307-0.758; *p* = 0.002). What’s more, univariate analysis displayed that the size of liver metastasis, number of liver metastases, synchronous metastases, extrahepatic metastases, and KRAS type were also related to PFS. Meanwhile, multivariate analysis showed the size of liver metastasis (HR, 1.317; 95%CI:1.131–1.532; *p* < 0.001), number of liver metastasis (HR, 2.042; 95%CI:1.243–3.354; *p* = 0.005), synchronous metastases (HR, 2.321; 95%CI, 1.237-4.357; *p* = 0.009) and extrahepatic metastases (HR,2.362; 95%CI: 1.298-4.297; *p* = 0.005) independently predicted worse PFS in unresectable CRLM patients.

**Table 3:** Univariate and multivariate Cox’s regression analysis for PFS.

Variables	Univariate analysis		Multivariate analysis	
	HR (95%CI)	*p* value	HR (95%CI)	*p* value
**Gender** (male/female)	0.947 (0.604 - 1.483)	0.811		
**Age**	1.006 (0.988 - 1.025)	0.526		
Primary tumor site				
Rectum	Ref	Ref	Ref	Ref
Left colon	1.345 (0.829 - 2.184)	0.23	1.062 (0.629 - 1.792)	0.822
Right colon	1.968 (1.111 - 3.484)	0.02	1.508 (0.816 - 2.788)	0.190
**Primary tumor resected**(yes/no)	0.699 (0.419 – 1.168)	0.172		
**Synchronous metastatic** (yes/no)	2.315 (1.308 - 4.094)	0.004	2.321 (1.237 – 4.357)	0.009
**Size of liver metastasis**	1.345 (1.179-1.535)	<0.001	1.317 (1.131 – 1.532)	<0.001
**Number of liver metastasis(>5/≤5)**	1.753 (1.083 - 2.835)	0.022	2.042 (1.243 – 3.354)	0.005
**Location of liver** **Metastases** (bilobar/unilobar)	1.121 (0.708 - 1.775)	0.626		
**Extrahepatic metastases** (yes/no)	2.505 (1.402 - 4.475)	0.002	2.362 (1.298 – 4.297)	0.005
**CEA level** (>5/≤5ng/ml)	1.504 (0.957 - 2.363)	0.077	1.548 (0.964 – 2.486)	0.070
**KRAS type** (Mutated/Wild)	1.262 (1.018 - 1.565)	0.034	1.551 (0.988 – 2.437)	0.057
**Treatment method** (DEB-TACE+systemic therapy/systemic therapy)	0.569 (0.372 - 0.871)	0.010	0.482 (0.307 – 0.758)	0.002

HR, hazard ratio; CI, confidence interval.

### Subgroup analysis

**Figure 3:**
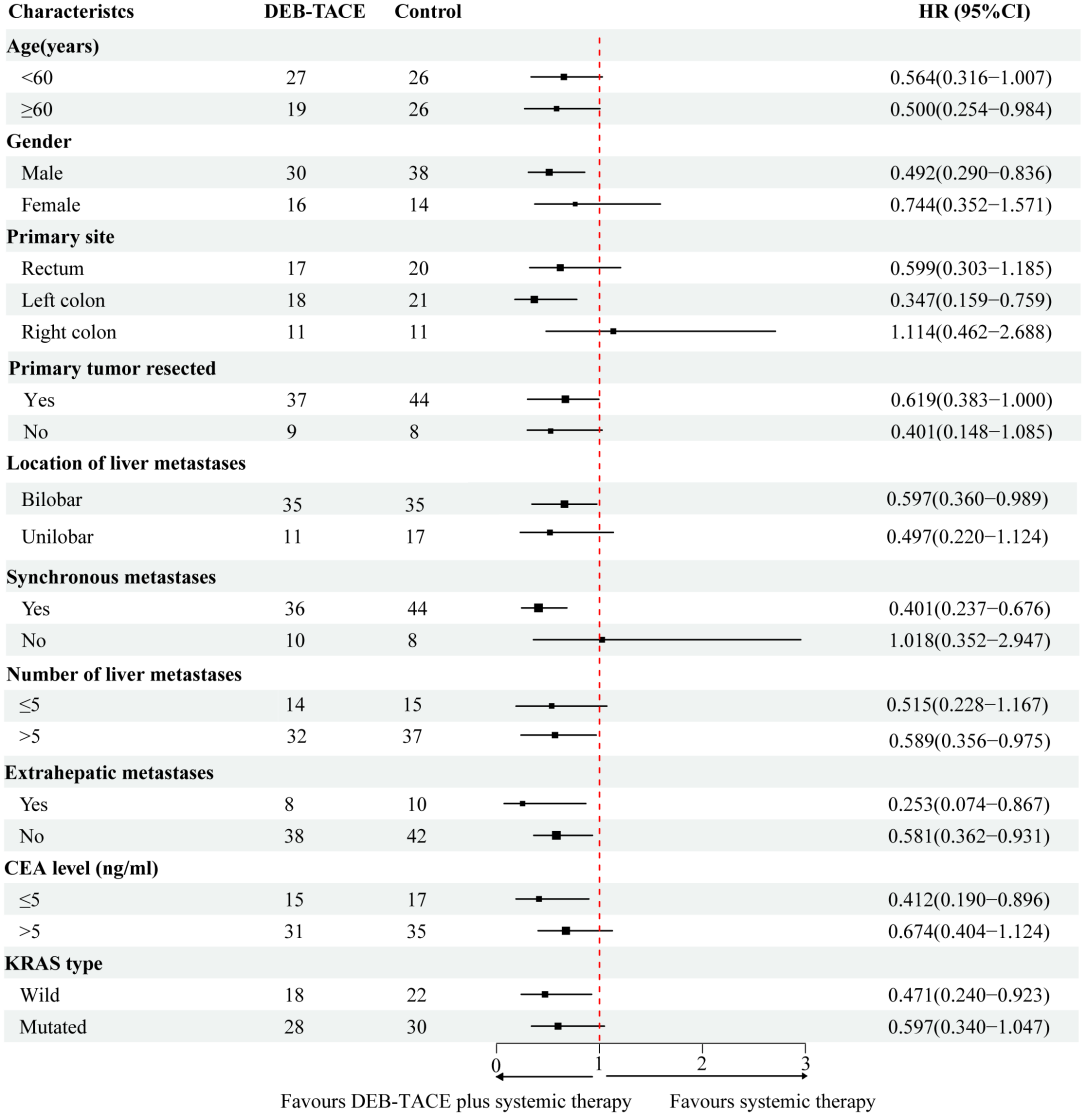
Forest plot depicting hazard ratios of the control group and the DEB-TACE group.

In the subgroup analysis, DEB-TACE plus systemic therapy had consistent beneficial effects on PFS across almost all tested subgroups. **Figure 3** presents the HR of the treatment group and the control group according to the patient’s clinical characteristics and treatment results. Nevertheless, only the subgroup of patients age ≥ 60, male, left colon, synchronous metastasis, bilobar, number of liver metastasis > 5, extrahepatic metastases, non-extrahepatic metastases, CEA level < 5 (ng/ml), and KRAS wild-type were observed statistically significant. In addition, multivariate analysis of the two groups was conducted separately to determine prognostic factors for the specific scheme. In the DEB-TACE group, the size of liver metastasis (*p* < 0.001), number of liver metastases > 5 (*p* = 0.025), extrahepatic metastases (*p* = 0.015), and CEA level > 5 (ng/ml) (*p* = 0.020) were significant risk factors for PFS. In the control group, synchronous metastatic (*p* = 0.015), extrahepatic metastases (*p* = 0.034), and KRAS type (*p* = 0.022) were related to the PFS (**Table 4**).

**Table 4:** Multivariate analysis by treatment modality for PFS.

Variables	DEB-TACE + systemic therapy		systemic therapy alone	
	HR (95%CI)	*p* value	HR (95%CI)	*p* value
Primary site				
Rectum	Ref	Ref	Ref	Ref
Left colon	1.055 (0.464 - 2.401)	0.898	1.490 (0.650 - 3.418)	0.347
Right colon	2.674 (1.040 - 6.870)	0.041	1.571 (0.615 - 4.012)	0.345
**Synchronous metastatic** (yes/no)	0.901 (0.355 - 2.287)	0.827	4.292 (1.323 - 13.927)	0.015
**Size of liver metastasis**	1.652 (1.256 - 2.173)	<0.001	1.182 (0.953 - 1.466)	0.128
**Number of liver metastasis(>5/≤5)**	2.431 (1.116 - 5.296)	0.025	1.527 (0.755 - 3.087)	0.238
**Extrahepatic metastases** (yes/no)	3.575 (1.279 - 9.989)	0.015	2.490 (1.074 - 5.773)	0.034
**CEA level** (>5/≤5ng/ml)	2.580 (1.160 - 5.740)	0.020	0.936 (0.479 - 1.831)	0.847
**KRAS type** (Wild/Mutated)	2.201 (0.983 - 4.923)	0.055	2.184 (1.119 - 4.264)	0.022

HR, hazard ratio; CI, confidence interval.

### Adverse events

Adverse events (AEs) related to additional DEB-TACE treatments in the DEB-TACE group are shown in **Table 5**. No death events related to the treatment occurred in our study. Among 146 DEB-TACE procedures, the most common AEs included vomiting/nausea (76.7%), poor appetite (71.9%), and abdominal distension (64.4%). No grade 4 or 5 toxicities were observed related to DEB-TACE procedures. Out of the total patients, one patient (0.7%) experienced severe vomiting (grade 3), and two patients (1.4%) had severe fever (grade 3). The remaining adverse events were generally mild to moderate and were successfully managed with symptomatic treatment, allowing for timely administration of subsequent chemotherapy combined with DEB-TACE. There was no significant difference in the occurrence of common grade ≥ 3 AEs associated with systemic chemotherapy, such as pancytopenia, hepatic and renal dysfunction, gastrointestinal reaction, and asthenia, between the two groups (*p* > 0.05) (**Supplementary Figure**). Chemotherapy delays due to toxicity were reported in 11 patients from the DEB-TACE group and 10 patients from the control group.

**Table 5:** Adverse events associated with DEB-TACE (146 procedures).

Adverse Events	All Events	CTCAE Grade			
		1	2	3	> 3
**Vomiting/Nausea**	112 (76.7%)	88 (60.3%)	23 (15.7%)	1 (0.7%)	0
**Poor appetite**	105 (71.9%)	58 (39.7%)	47 (32.2%)	0	0
**Abdominal distension**	94 (64.4%)	70 (47.9%)	24 (16.4%)	0	0
**Fatigue**	85 (58.2%)	54 (37.0%)	31 (21.2%)	0	0
**Abdominal pain**	54 (37.0%)	34 (23.3%)	20 (13.7%)	0	0
**Fever**	33 (22.6%)	17 (11.6%)	14 (9.6%)	2 (1.4%)	0
**Hyperbilirubinemia**	17 (11.6%)	12 (8.2%)	5 (3.4%)	0	0

CTCAE, Common Terminology Criteria for Adverse Events.

## Discussion

Although systemic chemotherapy remains the predominant treatment scheme for unresectable CRLM, its high risk of tumor recurrence makes it urgent to find an effective treatment to improve the therapeutic effect. Our research revealed that the addition of DEB-TACE as first-line therapy could prolong PFS in patients with unresectable CRLM, and similar findings were observed for liver-PFS. In addition, the DEB-TACE procedure was well tolerated with limited adverse effects from the combined treatment approach.

DEB-TACE combined with systemic therapy has shown promising results as the first-line treatment for unresectable CRLM. Robert C. G et al. (17) found that patients with unresectable CRLM receiving systemic chemotherapy combined with DEB-TACE as first-line therapy had a longer median PFS than patients receiving systemic therapy alone (15.3 months vs 7.6 months), which was similar to our results. It is worth noting that the combination therapy in their study showed a more remarkable improvement in median PFS than in ours, which could be attributed to baseline differences in the number of extrahepatic metastases between the two groups. In Phase II, a single-arm study, DEB-TACE in combination with systemic chemotherapy resulted in a median PFS of 10.8 months for unresectable CRLM as first-line therapy (18), which was shorter than the 12.1 months observed in our study. This discrepancy could be due to the smaller sample size in their study and potential differences in patient ethnicity. However, despite these differences, their study reported a high objective response rate of 73.2% and a resectable conversion rate of 33%, indicating the promising potential of DEB-TACE combined with mFOLFOX6 in unresectable patients with CRLM. The increased effectiveness may be attributed to DEB-TACE not only destroying metastases by blocking the vasculature of liver metastasis and local drug cytotoxicity, but also activating anti-tumor immunity through the release of tumor antigens from dead tumor cells (19, 20).

HAIC and TARE, local treatments for liver cancer, are also considered alternative options for patients with unresectable CRLM. Pak LM et al. (21) discovered that combining HAIC with systemic chemotherapy as the first-line treatment for CRLM patients resulted in a median PFS of 13 months, which was similar to our study. However, the technology required for HAIC pump implantation in HAIC treatment is more complex compared to DEB-TACE operation, presenting challenges to the widespread adoption of HAIC in China. By analyzing the results of three multicentre randomized trials (FOXFIRE, SIRFLOX, and FOXFIRE-Global), Wasan HS et al. (22) determined that the addition of TARE to FOLFOX chemotherapy regimen as the first-line treatment in patients with CRLM did not improve PFS or OS. Therefore, early use of TARE in combination with chemotherapy in unresectable CRLM is not recommended.

Our findings indicated that there was no significant difference in the rate of liver resectability between the two groups. However, it is noteworthy that despite the higher PFS observed in the DEB-TACE group, this did not translate into a higher hepatectomy rate compared to the previous results (23, 24). The reason for the analysis was that the dominant factors for the unresectability of patients were contribution to the number of liver metastases rather than their size. This implies that even if the tumor response rate were to improve, resection would still not be feasible. In addition, for approximately 40% of patients with liver metastases, hepatectomy was usually considered only when all areas of the lesion could be resected entirely. Hence, the likelihood that an improvement in liver response rate would affect resection was generally low (25). Moreover, although there was no reliable evidence of worse surgical efficacy or more severe complications after the DEB-TACE operation, liver surgeons might be reluctant to perform surgery after the DEB-TACE procedure, but the significance was uncertain.

Cox regression analysis revealed that both the size and number of liver metastases were identified as independent risk factors for PFS. This finding suggests that larger tumor sizes and a greater number of metastases pose challenges in the treatment of metastatic tumors (26). Interestingly, our study did not find tumor location to be a prognostic factor for PFS, which contrasts with previous findings (27, 28). This discrepancy could potentially be attributed to the ability of DEB-TACE procedures to target multiple hepatic lobes, potentially mitigating the impact of tumor location on PFS. Notably, He JH et al. (29) also observed that primary tumor location did not independently affect PFS in patients with CRLM undergoing first-line therapy, aligning with our study results. Conversely, Peter Gibbs et al. (30) reported that right colon primary site tumors were associated with poorer prognosis in CRLM patients, possibly potentially influenced by variations in treatment approaches and patient inclusion criteria.

Our study noted that synchronous liver metastasis was associated with poor PFS, consistent with the results reported in previous studies (31, 32). Synchronous metastasis might represent a more disseminated disease than metachronous metastasis. The diffusion trend might lead to early recurrence and poor prognosis in CRLM patients with synchronous metastases. However, from the results of separate multivariate analyses of the DEB-TACE group, there was no statistically significant difference between synchronous liver metastases and poor PFS. These outcomes might be attributed to the embolic effect of DEB-TACE operations on liver metastases and the cytotoxic effects of chemotherapeutic drug release on metastases. This suggested that the effect on the disseminated characteristics of liver metastases was reduced with DEB-TACE operations. In addition, this study illustrated that extrahepatic metastasis was a risk factor for PFS. Extrahepatic metastases might indicate a more aggressive tumor, more significant metastatic burden, and more challenging to control tumor growth, which could affect PFS in patients with unresectable CRLM.

Similar to other studies, DEB-TACE related toxicities were nausea/vomiting, poor appetite, bloating, fatigue, abdominal pain, fever, and hyperbilirubinemia, which were predominantly medically manageable. Common grade ≥ 3 AEs associated with chemotherapy were no significant difference between the two groups. This demonstrated that localized DEB-TACE therapy did not induce systemic toxicity to affect systemic treatment and concurrent irinotecan was delivered with this technique. None of the patients in both groups withdrew from treatment because of adverse events. Consequently, these results suggested that the combined treatment of DEB-TACE with systemic chemotherapy was effective and safe for patients with unresectable CRLM as the first-line treatment.

The limitations of the present study were as follows. First, due to the retrospective character of our research, it introduced potential selection and time-dependent assessment biases. Secondly, the limited population size of this study resulted in the restriction of its power. Finally, due to the relatively short period of follow-up time, this study did not achieve the median OS date. Therefore, further convincing prospective randomized controlled studies are required to confirm the safety and effectiveness of DEB-TACE combined with systematic therapy as first-line treatment in unresectable CRLM patients.

In conclusion, DEB-TACE combined with systemic therapy may improve progression-free survival and disease control rate outcomes over systemic therapy alone as a first-line treatment for unresectable CRLM patients. The addition of DEB-TACE operation did not negatively impact the administration of chemotherapy, and the adverse events profile was expected and manageable. This combination therapy as first-line treatment appears to be promising for the treatment of patients with unresectable.

## Data availability statement

The original contributions presented in the study are included in the article/**Supplementary Material**. Further inquiries can be directed to the corresponding authors.

## Ethics statement

The studies involving humans were approved by Union Hospital, Tongji Medical College, Huazhong University of Science and Technology. The studies were conducted in accordance with the local legislation and institutional requirements. Written informed consent for participation in this study was provided by the participants’ legal guardians/next of kin. Written informed consent was not obtained from the individual(s), nor the minor(s)’ legal guardian/next of kin, for the publication of any potentially identifiable images or data included in this article because of the nature of the retrospective study.

## Author contributions

FW: Conceptualization, Data curation, Formal analysis, Writing – original draft, Writing – review & editing, Investigation, Software. LC: Conceptualization, Data curation, Investigation, Writing – review & editing. CB: Conceptualization, Formal analysis, Investigation, Writing – review & editing. YC: Project administration, Software, Writing – review & editing. JW: Formal analysis, Writing – review & editing. GZ: Conceptualization, Data curation, Formal analysis, Software, Writing – original draft, Writing – review & editing. CZ: Conceptualization, Data curation, Formal analysis, Writing – original draft, Writing – review & editing.
