# Supplementary material for: Drug-eluting beads transcatheter arterial chemoembolization combined with systemic therapy versus systemic therapy alone as first-line treatment for unresectable colorectal liver metastases
Source: Front Oncol. 2024 Apr 24;14:1338293. doi: 10.3389/fonc.2024.1338293 (PMC11076665; doi:10.3389/fonc.2024.1338293)
Supplement: Supplementary file 1 [file Table_1.docx]

Supplementary Material

**Supplementary Table** Systemic therapy related adverse events ≥ grade 3 in two groups

| **Adverse Events** | **DEB-TACE (n=46)** | **Control (n=52)** | ***p* value** |
| --- | --- | --- | --- |
| **Pancytopenia** | 3 (6.5%) | 4 (7.7%) |  |
| **Hepatic and renal dysfunction** | 3 (6.5%) | 1 (1.9%) |  |
| **Hemorrhage** | 1 (2.2%) | 1 (1.9%) |  |
| **Gastrointestinal reaction** | 2 (4.3%) | 3 (5.8%) |  |
| **Wound infection** | 1 (2.2%) | - |  |
| **Hypertension** | - | 1 (1.9%) |  |
| **Asthenia** | 3 (6.5%) | - |  |
| **Total** | 11 (23.9%) | 10 (19.2%) | 0.573 |
